# Correlation of Interleukin-17-Producing Effector Memory T Cells and CD4^+^CD25^+^Foxp3 Regulatory T Cells with the Phosphate Levels in Chronic Hemodialysis Patients

**DOI:** 10.1155/2014/593170

**Published:** 2014-01-16

**Authors:** Cheng-Lin Lang, Min-Hui Wang, Kuan-Yu Hung, Sung-Hao Hsu, Chih-Kang Chiang, Kuo-Cheng Lu

**Affiliations:** ^1^Department of Internal Medicine, Cardinal Tien Hospital, Yonghe Branch 23445, Taiwan; ^2^Division of Nephrology, Department of Internal Medicine, Cardinal Tien Hospital, School of Medicine, Fu-Jen Catholic University, 362 Chung-Cheng Road, Hsin-Tien District, New Taipei City 23148, Taiwan; ^3^Division of Nephrology, Department of Internal Medicine, National Taiwan University Hospital, College of Medicine, National Taiwan University, Taipei 10002, Taiwan; ^4^Institute of Zoology, College of Life Science, National Taiwan University, Taipei 10002, Taiwan

## Abstract

*Background and Objectives*. Hyperparathyroidism and hyperphosphatemia contribute to the inflammatory effects in chronic hemodialysis (HD) patients. Interleukin-17-producing CD_4_^+^ effector memory T (Th17) cells and CD4^+^CD25^+^Foxp3 regulatory T (Treg) cells both play critical roles in immune activation and inflammation. We investigated the relationship between the Treg and Th17 cells and the phosphate level in chronic HD patients. *Methods*. 105 patients aged ≥35 years on chronic HD over 3 months were enrolled. The peripheral blood mononuclear cells were collected, cultured, and stimulated by phytohemagglutinin-L, phorbol myristate acetate, and ionomycin at different time points for T cell differentiation. *Results*. The T cell differentiation was as follows: Th17 cells (mean ± standard deviation (SD): 25.61% ± 10.2%) and Treg cells (8.45% ± 4.3%). The Th17 cell differentiation was positively correlated with the phosphate and albumin levels and negatively correlated with age. The Treg cell differentiation was negatively correlated with albumin level and age. In the nondiabetes group (*n* = 53), the Th17 cell differentiation was predominantly correlated with the phosphate and iPTH (intact parathyroid hormone) levels as well as the dialysis vintage. *Conclusion*. Higher phosphate and iPTH levels and longer dialysis duration may increase Th17 cell differentiation, especially in the nondiabetic chronic HD patients.

## 1. Introduction

The number of patients with end-stage renal disease (ESRD) is growing in developed countries, and hemodialysis (HD) is a major renal replacement therapy in these patients. Patients on HD have significant immune dysregulation as compared with the general population and, subsequently, have a high susceptibility to infection and high incidence of malignancy and cardiovascular disease [1]. Uremia and its treatment cause immune alterations in HD patients [2]. Several factors influence the immunity of these patients, such as uremic toxin, malnutrition, chronic inflammation, vitamin D-parathyroid hormone axis alternation, and therapeutic dialysis modalities [3–5].

CD_4_^+^ T cells regulate several immune responses and inflammatory processes. Currently, CD_4_^+^ T cells can be divided into several subsets, including Th1, Th2, Th17, and regulatory T cells (Treg) in the adaptive immune system [6]. A subset of interleukin (IL)-17-producing Th17 cells, distinct from the Th1 and Th2 cells, have an important role in the development of tissue inflammation and autoimmune disease such as rheumatoid arthritis and allergen-specific responses [7, 8]. The Th17 cell expresses a transcription factor, retinoic acid-related orphan receptor-*γ*t (ROR*γ*t), and plays a crucial role in the induction of autoimmune disease and inflammation [9]. The combination of acute phase protein IL-6 and transforming growth factor (TGF)-*β* induces the differentiation of Th17 cells from naïve T cells. Another small proportion of CD_4_^+^ T cells develop into Treg cells by their coexpression of high levels of surface CD25 and intracellular forkhead/winged helix transcription factor (Foxp3) [10, 11]. The Treg cells have an anti-inflammatory role and control autoimmune diseases by releasing IL-10 and TGF-*β* [12]. Therefore, the balance between Th17/Treg cells plays an important role in the inflammation in the adaptive immune system.

In chronic kidney disease-mineral and bone disorders (CKD-MBD), hyperphosphatemia plays a major role, causing secondary hyperparathyroidism, calcium and vitamin D derangements, vascular calcification, and several mineral bone disorders [13]. Increased serum calcium-phosphate product and mineral metabolism disorders may induce cardiovascular calcification [14]. Accelerated atherosclerosis and vascular calcification are involved in the pathogenesis of cardiovascular disease in CKD patients. Apart from traditional risk factors such as male gender, diabetes, ageing, dyslipidemia, and smoking, nonclassical risk factors such as malnutrition, microinflammation, hyperphosphatemia, hyperparathyroidism, and oxidative stress are important in the pathogenesis of cardiovascular disease in dialysis patients [15, 16]. Therefore, dietary phosphate control and use of phosphate binders are important in HD patients with hyperphosphatemia.

Studies have shown that, in dialysis patients, Th17 cells increase, but Treg cells decrease [17, 18]. From this perspective, whether T cell differentiation is correlated to factors in chronic HD patients and whether the phosphate levels influence T cell differentiation remain unclear. Hence, this study aimed at investigating the Th17/Treg cell differentiation in chronic HD patients and the correlation between Th17/Treg cell imbalance and serum biochemistry results.

## 2. Materials and Methods

### 2.1. Study Design and Populations

This study was conducted at a dialysis clinic in a regional hospital in Taiwan. In total, 105 patients aged ≥35 years on chronic HD for at least 3 months were enrolled. Patients with concurrent systemic infection or malignancy and those who were administered immunosuppressive medications known to interfere with the immune system were excluded from this study. Medications, when necessary, included antihypertensive treatments, oral hypoglycemic drugs or insulin therapy, antidyslipidemia medication, laxatives, and/or coronary vasodilator. In the subanalysis, we divided the 105 patients into 2 groups, the diabetes and nondiabetes groups due to the possible immune alteration by the glycemic control.

Dialysis was performed with bicarbonate dialysate and a high-flux polysulfone membrane dialyzer without reprocessing. Each hemodialysis session was performed for 3-4 h using the dialyzer with a blood flow rate of 200–300 mL/min and a dialysate flow of 500 mL/min. All patients gave informed consent for this study, and the study was reviewed and approved by the Human and Ethics Committee of the Cardinal Tien Hospital, Yonghe Branch, Taiwan (IRB-A101002).

### 2.2. Isolation and Culture Conditions of Peripheral Blood Mononuclear Cells

Blood samples (10 mL) were collected just before the second dialysis session of the week (midweek predialysis). The peripheral blood mononuclear cells were isolated from the buffy coats using Ficoll-Paque (Pharmacia Biotech AB, Uppsala, Sweden) density gradient centrifugation. Cells were cultured at 2 × 10^6^ cells/mL in RPMI-1640 (Gibco BRL, Paisley, Scotland) medium with a supplement of 10% fetal calf serum (Biochrome KG) and antibiotics (100 IU/mL penicillin, 100 *μ*g/mL streptomycin). Peripheral blood mononuclear cells were primarily stimulated for 4 h using 20 ng/mL phorbol myristate acetate (PMA, Sigma) and 1 *μ*M/mL ionomycin (Sigma) in the presence of the intracellular cytokine transport inhibitor GolgiStop (1 *μ*L/mL, BD Biosciences, San Jose, CA, USA). The cells were all cultured in a humidified incubator at 37°C and 5% CO_2_. The cells were then stained to identify surface and intracellular cytokine markers.

### 2.3. Antibodies

All antibodies were labeled by different fluorescence. The monoclonal antibody used to detect the surface antigen was FITC-labeled anti-CD4 (Beckman). Antibodies used for the intracellular stain were ECD-labeled anti-CD25 (Beckman), PE-labeled anti-IL17*α* (eBioscience, San Diego, CA, USA), and PECy7-labeled anti-FoxP3 (eBioscience).

### 2.4. Intracellular Cytokine Staining

After stimulation with PMA and ionomycin, as previously mentioned, the aliquots with 10^5^ cells/tube were used for intracellular cytokine staining. To detect Th17 cells, the cells were incubated with FITC anti-CD4 at 4°C for 20 min and stained with PE-labeled anti-IL17*α* after fixation and permeabilization, according to the manufacturer's instructions. To detect Treg cells, the cells were incubated with FITC anti-CD4 and ECD-labeled anti-CD25 for surface staining. After fixation and permeabilization, the cells were stained with PECy7-labeled anti-FoxP3. The cells were resuspended and washed with phosphate buffer saline and analyzed with FACS Calibur (Becton Dickinson, Franklin Lakes, NJ, USA) using CellQuest software (Becton Dickinson). Isotype controls were used as compensation controls, and antibody specificity was confirmed.

### 2.5. Biochemistry Analysis

Biochemical and hematological parameters were obtained by midweek predialysis in chronic HD patients. Hemoglobin and hematocrit levels were evaluated using an XT100i automated chemistry analyzer (Sysmex, Japan). Prehemodialysis blood urea nitrogen, creatinine, total calcium, serum phosphate, and serum albumin levels were evaluated using an LX20 automated analyzer (Beckman Coulter, CA, USA). Kt/V, a marker of dialysis efficiency, was determined according to the Gotch procedure.

### 2.6. Statistical Analysis

Continuous variables were expressed as mean ± standard deviation, and categorical values were expressed as percentages. The differences between the diabetes and nondiabetes groups were analyzed by independent *t*-tests. Pearson correlations were derived to evaluate the possible correlations between the biological markers and continuous variables. Significance was defined by *P* values <0.05. Statistical analyses were performed with the Statistical Package for the Social Sciences, version 17.0 (SPSS Inc., Chicago, IL, USA).

## 3. Results

### 3.1. General Characteristics of the Study Subjects

The mean age of patients was 65.72 ± 12.7 years, and 52 of the 105 patients had diabetes. The causes of ESRD in the diabetes group were diabetes (*n* = 44), hypertension (*n* = 6), chronic glomerulonephritis (*n* = 1), and heart failure (*n* = 1). In the nondiabetes group, the causes of ESRD were hypertension (*n* = 30), chronic glomerulonephritis (*n* = 12), obstructive nephropathy (*n* = 4), autosomal dominant polycystic disease (*n* = 3), systemic lupus nephritis (*n* = 3), and heart failure (*n* = 1). More patients in the nondiabetes group were infected with the hepatitis C virus than those in the diabetes group. There was no statistically significant difference in age, sex, dialysis vintage, or hepatitis B virus infection between the 2 groups. The baseline characteristics data are shown in Table 1.

### 3.2. The Biochemistry and T Cell Differentiation Results of the Study Population

The T cell differentiation of the total population was as follows: Th17 cells (25.61% ± 10.2%) and Treg cells (8.45% ± 4.3%). The total white cell count was greater in the diabetes group than in the nondiabetes group (*P* < 0.05), but no statistically significant difference was found in the lymphocyte count (*P* = 0.06). The glucose and triglyceride levels were higher in the diabetes group. However, there were no statistically significant differences in the hemoglobin, albumin, intact parathyroid hormone (iPTH), calcium, phosphate, alkaline phosphate, and C-reactive protein (CRP) levels, as well as Th17 cell and Treg cell differentiation between the 2 groups (Table 2).

### 3.3. Th17 and Treg Cell Frequencies Are Correlated with Phosphorus Level, Age, and Albumin Level

**Figure 1:**
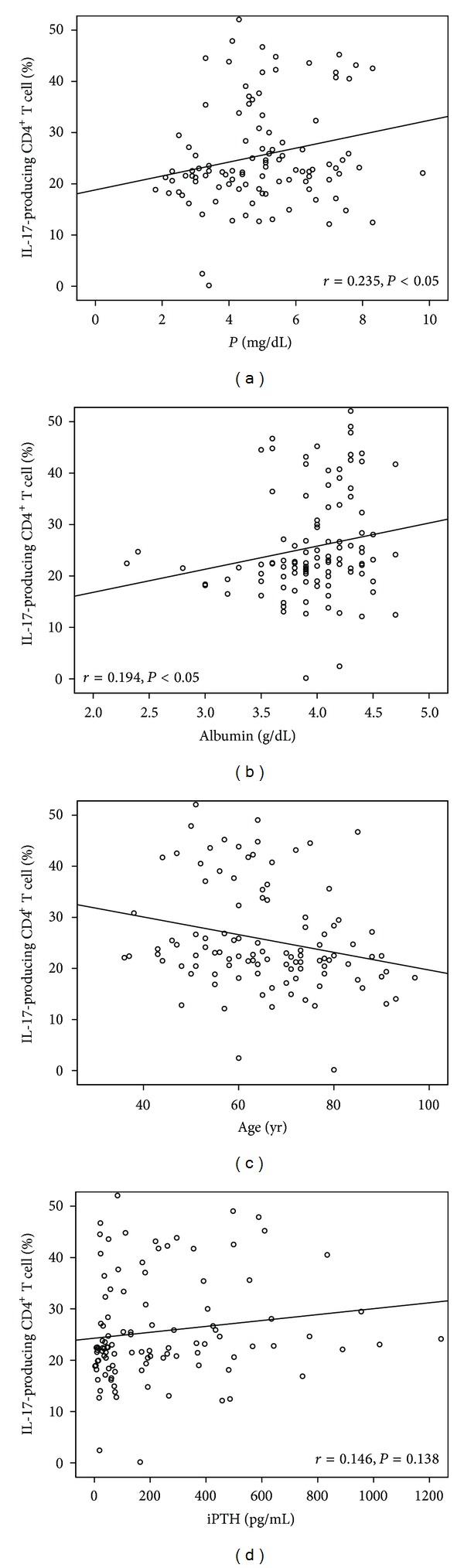
IL-17-producing CD_4_^+^ T cell differentiation was correlated with phosphorus (a), albumin (b), age (c), and intact parathyroid hormone (iPTH) (d).

**Figure 2:**
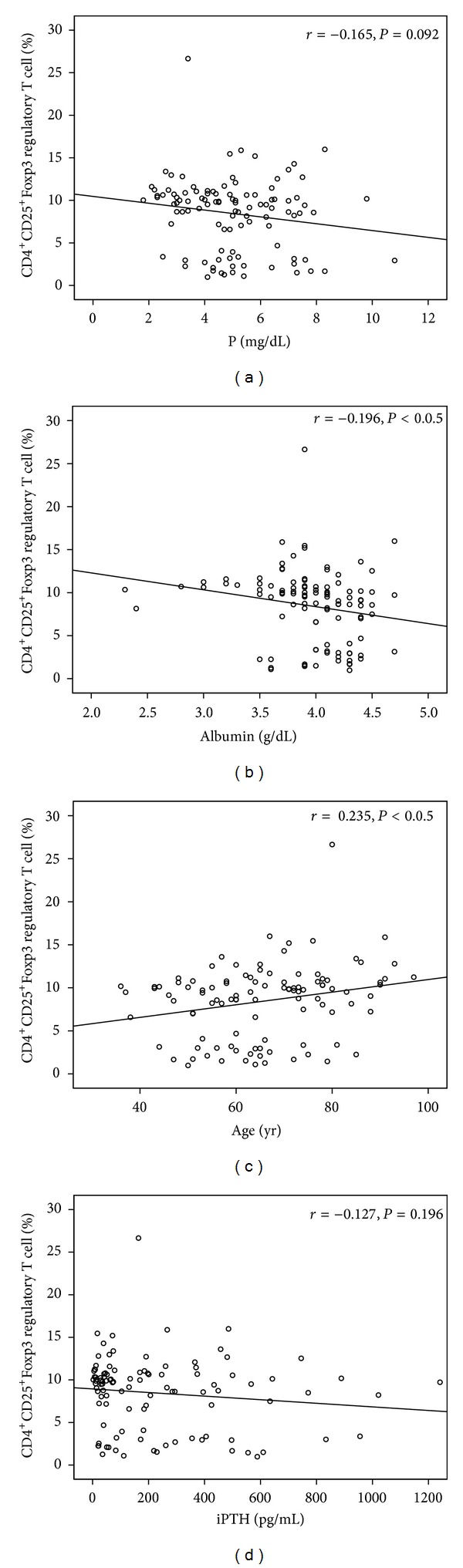
CD4^+^CD25^+^Foxp3 T cell differentiation was correlated with phosphorus (a), albumin (b), age (c), and parathyroid hormone (d).

The Th17 cell differentiation was correlated with the phosphate level, age, and albumin level, but not with the iPTH level (Figure 1). The Treg cell differentiation was also correlated with age and the albumin level, but not with the phosphate level or iPTH level (Figure 2). The Th17 cell and Treg cell differentiation was not correlated with the glucose, hemoglobin, calcium, alkaline phosphate, or CRP levels.

### 3.4. Th17 Cell Differentiation Was Significantly Correlated with Phosphate Levels, iPTH Levels, and Dialysis Vintage in Patients without Diabetes

**Figure 3:**
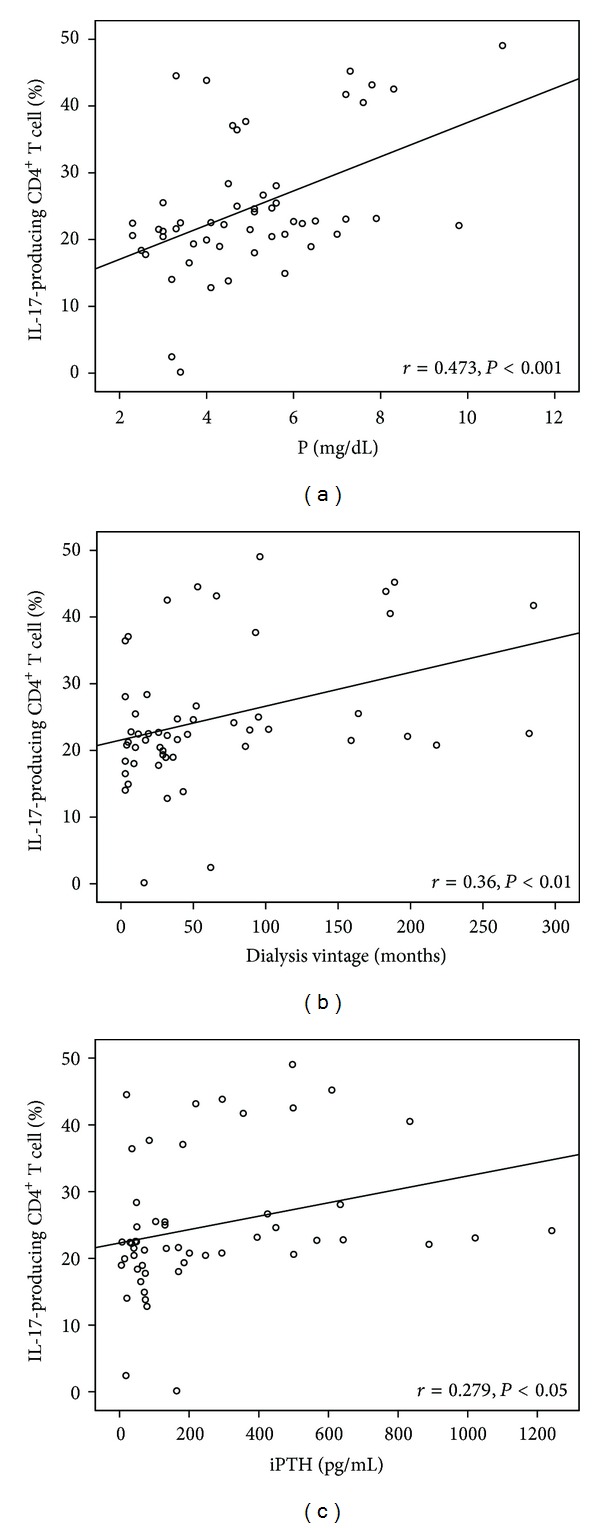
The Th17 cell differentiation correlated with phosphorus level (*P* < 0.001, (a)), iPTH level (*P* < 0.05, (c)), and dialysis vintage (*P* < 0.01, (b)).

In chronic HD patients without diabetes, the Th17 cell differentiation was predominantly correlated with the phosphate level, iPTH level, and dialysis vintage. Patients with higher serum phosphate levels had more severe secondary hyperparathyroidism, longer dialysis vintage, and more Th17 cells (Figure 3). However, no such correlation was noted in the diabetes group.

### 3.5. Treg Cell Differentiation Was Positively Correlated with Age but Negatively Correlated with the Phosphate Level and Dialysis Vintage

**Figure 4:**
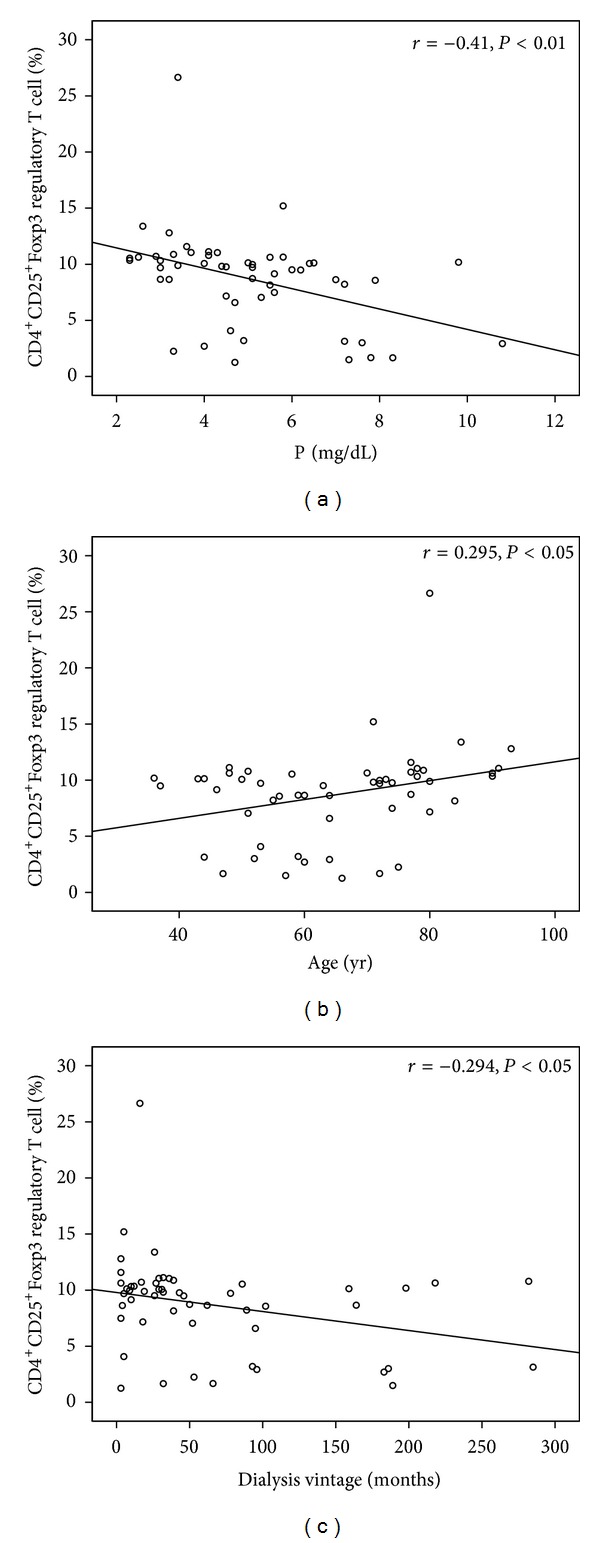
The Treg cell differentiation correlated with phosphorus level (*P* < 0.01, (a)), dialysis vintage (*P* < 0.05, (c)), and albumin level (*P* < 0.05, (b)).

In chronic HD patients without diabetes, Treg cell differentiation was found to be correlated with age but negatively correlated with the phosphate level and dialysis vintage. Patients without diabetes who were older and had lower serum phosphate levels and shorter duration of HD had more Treg cells (Figure 4).

## 4. Discussion

The results of this study indicate that Th17 cells are increased whereas Treg cells are decreased in chronic HD patients. There was a significant correlation between the Th17/Treg differentiation and the phosphate level, albumin level, and age. In the chronic HD patients without diabetes, the correlation was more significant between the Th17 cells and the phosphate level, iPTH level, and dialysis vintage. The Treg cells had a negative linear correlation with the phosphate level and dialysis vintage. These results suggest that Th17 cell and Treg cell differentiation are related to the serum phosphate level, nutritional status, and the duration of dialysis in chronic HD patients.

For many years, the heterogeneity of CD_4_^+^ T helper (Th) cells has been limited to Th1 and Th2 cells, which are considered to be responsible not only for different types of protective responses, but also for the pathogenesis of many disorders [19]. Th1 cells, which produce interferon-*γ* and tumor necrosis factor-*α*, are effective in eliminating intracellular pathogens and have a proinflammatory and proatherogenic role in atherosclerosis [20–23]. Th2 cells, with IL-4 as the major cytokine, are important for the production of immunoglobulin, clearance of extracellular organisms, and protection against helminthes; in addition, they are responsible for the pathogenesis of allergic diseases [24, 25]. In a previous study, we found that Th2 differentiation was correlated with age and serum vitamin D levels in chronic HD elderly patients, and treatment with activated vitamin D in secondary hyperparathyroidism patients enhanced Th2 differentiation [26]. In recent years, the importance of the role of Th17 and Treg cells in the adaptive immune system has become more evident. The Th17 cell is a key player in the pathogenesis of autoimmune disease, while the Treg cell inhibits excessive effector T cell response. Excessive Th17 function or increased Th17 cell numbers and defects in Treg function or reduced Treg cell numbers may trigger an inflammatory process [27]. Because of the reciprocal developmental pathway for the generation of Th17 and Treg cells, and their opposing effects, Th17/Treg subsets may have evolved to induce or regulate tissue inflammation, parallel to the dichotomy of Th1/Th2 T cell subsets. Any imbalance in the Th17/Treg ratio with increased Th17 cells and/or decreased Treg cells may induce local tissue inflammation. A Th17/Treg functional imbalance exists in uremic patients and is associated with the development of acute cardiovascular events, myocardial injury, and microinflammation [17, 28]. In ESRD patients, the CD4^+^CD25^+^Foxp3 T cells are overactivated but functionally impaired [29]. In the chronic HD patients of the present study, Th17 cell differentiation was greater than that of Treg cells, and our results are consistent with those of previously published studies. In the diabetes group, there were a relatively higher number of Th17 cells and lower number of Treg cells than those in the nondiabetes group. In the study by Campean et al. [30] higher inflammatory status of coronary lesions as well as involvement of the CD40–CD154 signaling cascade in CRF patients was reported, especially in cases of calcified atherosclerotic lesions. In our study, the inflammatory marker—CRP level—was also relatively higher in diabetes patients than in patients without diabetes, but there was no statistically significant difference between the 2 groups, possibly because of the small sample size.

Regarding CKD-MBD, hyperphosphatemia, hypercalcemia, and hyperparathyroidism contribute to the development of vascular calcification and cardiovascular disease, especially in patients on dialysis for a long duration. As CKD progresses, the compensation of elevations in PTH and fibroblast growth factor-23 (FGF-23) and decreased levels of 1,25(OH)_2_D_3_ becomes inadequate and inappropriate, resulting in hyperphosphatemia, abnormal bone, and extraskeletal calcification. There is much evidence that strict phosphate control may improve the overall survival and reduce the incidence of cardiovascular events [31–33]. Phosphate toxicity due to excessive retention of phosphate in the body can cause a wide range of cellular and tissue injuries [34]. When hyperphosphatemia occurred in the HD patients, the serum FGF-23 level elevated, 1,25(OH)_2_D_3_ level decreased, and renin-angiotensin-aldosterone system activation, all may deteriorate the systemic inflammation. Besides, hyperphosphatemia causes secondary hyperparathyroidism also cause inflammation in chronic HD patients. The findings of this study suggest that the phosphate level is associated with Th17 cell differentiation, as a higher number of Th17 cells were noted in those with higher serum phosphate levels. PTH has been identified as a regulator in the immune system and regulated the inflammatory processes. Chronic PTH exposure in ESRD patients decreased T lymphocyte proliferation and changed the CD4/CD8 ratio [35]. In in vitro and in vivo models, PTH may induce IL-6 production by liver cells and osteoblast [16]. In primary hyperparathyroidism, the parathyroid glands contribute markedly to IL-6 production and elevate the serum IL-6 level [36]. Our previous study also demonstrated that increased serum iPTH level would enhance serum IL-6 production in HD patients [16]. Calcitriol treatment significantly attenuates inflammation and oxidative stress in HD patients with secondary hyperparathyroidism [35]. The serum vitamin D level is correlated with Th2 differentiation, and supplement/treatment with activated vitamin D in secondary hyperparathyroidism HD patients can enhance Th2 differentiation [26]. From this study, we noted that Th17 cell—a kind of inflammatory marker—positively correlated to the serum iPTH level in the HD patients without diabetes. In the Floege study, there is a linear trend between the phosphate, serum iPTH, and albumin level in chronic HD patients [33]. In these HD patients, higher serum iPTH level patients also had a higher serum phosphate level and albumin level. This is the reason that Th17 cell differentiation correlated to the serum phosphate, iPTH, and albumin level in our study.

We found the phosphate level to be a significant predictive factor for Th17 and Treg cell differentiation in patients without diabetes. In patients with type 2 diabetic nephropathy, the role of Th17 and Treg cells in the pathogenesis of diabetic nephropathy remains unclear [37]. The study by Ishimura et al. [38] reported that the risk factor for vascular calcification in dialysis patients with and without diabetes is different. Glycemic control and phosphate control are important for HD patients with and without diabetes, respectively, to avoid vascular calcification. In the present study, Th17 and Treg cell differentiation was not correlated with the phosphate level in the diabetes group, but the correlation was significant in the nondiabetes group. It is possible that HD patients with diabetes have multiple metabolic factors that may affect T cell differentiation before they initiate dialysis, including hyperglycemia, dyslipidemia, insulin resistance, advanced glycosylation end (AGE) products, or oxidative stress. In diabetes patients, the AGE or AGE-modified proteins would bind to the receptor for AGE on macrophages and T cells, stimulating synthesis and release of proinflammatory cytokines [37]. Hence, we were not able to identify the association between the phosphate or iPTH level and T cell differentiation in our HD patients with diabetes.

In the nephrology field, much evidence has shown that Th17 and Treg cells have an important role in the inflammatory process. In patients with acute coronary syndrome, the number of peripheral blood Th17 cells, Th17-related cytokines (IL-17, IL-6, and IL-23), and transcription factor (ROR*γ*t) levels are significantly increased, and the number of Treg cells, Treg-related cytokines (IL-10 and TGF-*β*1), and transcription factor (Foxp3) levels in the serum are decreased [39]. The Th17/Treg ratio is involved in inflammation control and may be important in the pathogenesis of plaque destabilization. Many chronic renal diseases have inflammation and infiltration of leukocytes, while Th17/Treg cell imbalance during renal injury is involved in the strong relationships between chemokines and T cell infiltration in the development of kidney disease [40]. The Th17/Treg cell ratio is a useful indicator of the severity of tissue injury and renal allograft dysfunction and for predicting the clinical outcome of acute T cell-mediated rejection [41]. Using calcineurin inhibitors after transplantation would influence the Th17/Treg cell imbalance in patients with renal dysfunction [42]. In children with primary nephrotic syndrome, Th17 and Treg cells are in a dynamic equilibrium, and these cells may be important in the development of renal tubulointerstitial lesions [43]. Therefore, finding a way to decrease inflammatory Th17 cells and increase anti-inflammatory Treg cells in clinical practice is challenging.

The present study had several limitations. Because of the current cross-sectional study design, long-term followup is needed to confirm if T cell differentiation changes after strict phosphate control. In addition, we only enrolled HD patients, and peritoneal dialysis or CKD patients who may have had different patterns of associations between the phosphate level and Th17/Treg cells were not included. Finally, cytokine analyses may be needed to strengthen our results, including Th17 cell-related cytokines (IL-17 and IL-6) and Treg cell-related cytokines (TGF-*β* and IL-10). Therefore, our results regarding Th17/Treg cell differentiation and phosphate level need to be confirmed by a prospective long-term follow-up study in the future.

## 5. Conclusion 

The ratio of Th17/Treg cells is imbalanced in chronic HD patients. Chronic HD patients with higher phosphate and albumin levels, as well as younger patients, have increased Th17 cells and decreased Treg cell differentiation. Th17 and Treg cell differentiation was predominantly correlated with phosphate, iPTH levels, and dialysis vintage in HD patients without diabetes.

## Figures and Tables

**Table 1:** Baseline clinical characteristics of subjects (mean ± SD) in the diabetes and nondiabetes groups.

	Total	Diabetes group	Nondiabetes group
Patients (*n*)	105	52	53
Age (years)	65.72 ± 13.7	66.35 ± 12.6	65.11 ± 14.8
Sex (M/F)	55/50	29/23	26/27
HBsAg (+/−)	8/97	4/48	4/49
Anti-HCV (+/−)	14/91	3/49	11/42*
Cause of end-stage renal disease	ADPKD: 3, CGN: 13, diabetes: 44, heart failure: 1, HTN: 36, obstructive nephropathy: 4, and SLE: 3	CGN: 1, diabetes: 44, heart failure: 1, and HTN: 6	ADPKD: 3, CGN: 12, HTN: 30, heart failure: 1, obstructive nephropathy: 4, and SLE: 3
Dialysis vintage (months)	55.3 ± 58.6	46.21 ± 37.5	64.25 ± 73.1

*P < 0.05 versus the diabetes group.

CGN: chronic glomerulonephritis; HTN: hypertension; ADPKD: autosomal dominant polycystic disease; SLE: systemic lupus nephritis.

**Table 2:** Biochemistry and flow cytometry results of subjects (mean ± SD) in the diabetes and nondiabetes groups.

	Diabetes group	Nondiabetes group
Patients (*n*)	52	53
Hemoglobin (g/dL)	10.43 ± 1.4	10.14 ± 1.8
Glucose (mg/dL)	155.4 ± 82.3	98.25 ± 22.6**
Albumin (g/dL)	4.02 ± 0.3	3.90 ± 0.5
Predialysis BUN (mg/dL)	60.62 ± 18.5	57.19 ± 16.8
Predialysis Cre (mg/dL)	9.59 ± 2.4	9.80 ± 2.6
Kt/V	1.62 ± 0.3	1.71 ± 0.3
Uric acid (mg/dL)	6.70 ± 1.4	6.68 ± 1.4
Triglyceride (mg/dL)	191.10 ± 137.9	130.53 ± 92.3**
Total cholesterol (mg/dL)	174.38 ± 42.4	170.79 ± 40.9
Alkaline phosphate (IU/L)	69.94 ± 30.8	63.32 ± 22.5
Total calcium (mg/dL)	9.43 ± 0.7	9.38 ± 0.9
Phosphate (mg/dL)	4.99 ± 1.6	5.04 ± 1.9
iPTH (pg/dL)	212.12 ± 228.2	250.59 ± 286.2
WBC counts (×1000/*μ*L)	6.80 ± 1.4	6.15 ± 1.8*
Lymphocyte count (×1000/*μ*L)	1.78 ± 0.5	1.55 ± 0.7
Th17 cell (%)	26.43 ± 10.1	24.82 ± 10.3
Treg cell (%)	8.19 ± 4.4	8.70 ± 4.2
CRP (mg/dL)	0.96 ± 2.0	0.57 ± 1.0

*P < 0.05 versus the diabetes group **P < 0.01 versus the diabetes group.

Cre: creatinine; iPTH: intact parathyroid hormone; WBC: white blood cell; CRP: C-reactive protein.

## References

[1] Eleftheriadis T, Antoniadi G, Liakopoulos V, Kartsios C, Stefanidis I (2007). Disturbances of acquired immunity in hemodialysis patients. *Seminars in Dialysis*.

[2] Girndt M, Sester U, Sester M, Kaul H, Kohler H (1999). Impaired cellular immune function in patients with end-stage renal failure. *Nephrology Dialysis Transplantation*.

[3] Sterling KA, Eftekhari P, Girndt M, Kimmel PL, Raj DS (2012). The immunoregulatory function of vitamin D: implications in chronic kidney disease. *Nature Reviews Nephrology*.

[4] Tzanno-Martins C, Futata E, Jorgetti V, Duarte AJS (2000). Restoration of impaired T-cell proliferation after parathyroidectomy in hemodialysis patients. *Nephron*.

[5] Vanholder R, de Smet R, Hsu C, Vogeleere P, Ringoir S (1994). Uremic toxicity: the middle molecule hypothesis revisited. *Seminars in Nephrology*.

[6] Zhu J, Paul WE (2008). CD4 T cells: fates, functions, and faults. *Blood*.

[7] Kolls JK, Lindén A (2004). Interleukin-17 family members and inflammation. *Immunity*.

[8] Stockinger B, Veldhoen M, Martin B (2007). Th17 T cells: linking innate and adaptive immunity. *Seminars in Immunology*.

[9] Ivanov II, McKenzie BS, Zhou L (2006). The orphan nuclear receptor ROR*γ*t directs the differentiation program of proinflammatory IL-17^+^ T helper cells. *Cell*.

[10] Chen W, Jin W, Hardegen N (2003). Conversion of peripheral CD4^+^CD25- naive T cells to CD4^+^CD25^+^ regulatory T cells by TGF-*β* induction of transcription factor Foxp3. *Journal of Experimental Medicine*.

[11] Wing K, Sakaguchi S (2010). Regulatory T cells exert checks and balances on self tolerance and autoimmunity. *Nature Immunology*.

[12] Sakaguchi S, Ono M, Setoguchi R (2006). Foxp3^+^CD25^+^CD4^+^ natural regulatory T cells in dominant self-tolerance and autoimmune disease. *Immunological Reviews*.

[13] Zheng CM, Lu KC, Wu CC, Hsu YH, Lin YF (2011). Association of serum phosphate and related factors in ESRD-related vascular calcification. *International Journal of Nephrology*.

[14] Cozzolino M, Brancaccio D, Gallieni M, Slatopolsky E (2005). Pathogenesis of vascular calcification in chronic kidney disease. *Kidney International*.

[15] Campean V, Neureiter D, Varga I (2006). Atherosclerosis and vascular calcification in chronic renal failure. *Kidney and Blood Pressure Research*.

[16] Lu K-C, Tseng C-F, Wu C-C (2006). Effects of calcitriol on type 5b tartrate-resistant acid phosphatase and interleukin-6 in secondary hyperparathyroidism. *Blood Purification*.

[17] Zhang J, Hua G, Zhang X, Tong R, Du X, Li Z (2010). Regulatory T cells/T-helper cell 17 functional imbalance in uraemic patients on maintenance haemodialysis: a pivotal link between microinflammation and adverse cardiovascular events. *Nephrology*.

[18] Libetta C, Esposito P, Sepe V (2010). Dialysis treatment and regulatory T cells. *Nephrology Dialysis Transplantation*.

[19] Annunziato F, Romagnani S (2009). Heterogeneity of human effector CD4^+^ T cells. *Arthritis Research and Therapy*.

[20] Hansson GK (2005). Mechanisms of disease: inflammation, atherosclerosis, and coronary artery disease. *The New England Journal of Medicine*.

[21] Hsieh C-S, Macatonia SE, Tripp CS, Wolf SF, O’Garra A, Murphy KM (1993). Development of TH1 CD4^+^ T cells through IL-12 produced by Listeria-induced macrophages. *Science*.

[22] Robertson A-KL, Hansson GK (2006). T cells in atherogenesis: for better or for worse?. *Arteriosclerosis, Thrombosis, and Vascular Biology*.

[23] Scharton TM, Scott P (1993). Natural killer cells are a source of interferon *γ* that drives differentiation of CD4^+^ T cell subsets and induces early resistance to Leishmania major in mice. *Journal of Experimental Medicine*.

[24] Bottomly K (1988). A functional dichotomy in CD4^+^ T lymphocytes. *Immunology Today*.

[25] Shinkai K, Mohrs M, Locksley RM (2002). Helper T cells regulate type-2 innate immunity in vivo. *Nature*.

[26] Lang CL, Wang MH, Hung KY, Chiang CK, Lu KC (2013). Altered molecular repertoire of immune system by renal dysfunction in the elderly: is prediction and targeted prevention in the horizon?. *EPMA Journal*.

[27] Kimura A, Kishimoto T (2010). IL-6: regulator of Treg/Th17 balance. *European Journal of Immunology*.

[28] Chen D, Huang X, Yang M, Gan H, Gunawan EJ, Tang W (2012). Treg/Th17 functional disequilibrium in Chinese uremia on hemodialysis: a link between calcification and cardiovascular disease. *Renal Failure*.

[29] Hendrikx TK, van Gurp EAFJ, Mol WM (2009). End-stage renal failure and regulatory activities of CD4^+^CD25bright^+^FoxP3^+^ T-cells. *Nephrology Dialysis Transplantation*.

[30] Campean V, Neureiter D, Nonnast-Daniel B, Garlichs C, Gross M-L, Amann K (2007). CD40-CD154 expression in calcified and non-calcified coronary lesions of patients with chronic renal failure. *Atherosclerosis*.

[31] Cannata-Andía JB, Fernández-Martín JL, Zoccali C (2008). Current management of secondary hyperparathyroidism: a multicenter observational study (COSMOS). *Journal of Nephrology*.

[32] Waheed AA, Pedraza F, Lenz O, Isakova T (2013). Phosphate control in end-stage renal disease: barriers and opportunities. *Nephrology Dialysis Transplantation*.

[33] Floege J, Kim J, Ireland E (2011). Serum iPTH, calcium and phosphate, and the risk of mortality in a European haemodialysis population. *Nephrology Dialysis Transplantation*.

[34] Razzaque MS (2011). Phosphate toxicity: new insights into an old problem. *Clinical Science*.

[35] Wu C-C, Chang J-H, Chen C-C (2011). Calcitriol treatment attenuates inflammation and oxidative stress in hemodialysis patients with secondary hyperparathyroidism. *Tohoku Journal of Experimental Medicine*.

[36] Safley SA, Villinger F, Jackson EH, Tucker-Burden C, Cohen C, Weber CJ (2004). Interleukin-6 production and secretion by human parathyroids. *Clinical and Experimental Immunology*.

[37] Wu C-C, Sytwu H-K, Lu K-C, Lin Y-F (2011). Role of T cells in type 2 diabetic nephropathy. *Experimental Diabetes Research*.

[38] Ishimura E, Okuno S, Taniwaki H (2008). Different risk factors for vascular calcification in end-stage renal disease between diabetics and nondiabetics: the respective importance of glycemic and phosphate control. *Kidney and Blood Pressure Research*.

[39] Cheng X, Yu X, Ding Y-J (2009). The Th17/Treg imbalance in patients with acute coronary syndrome. *Clinical Immunology*.

[40] Chung ACK, Lan HY (2011). Chemokines in renal injury. *Journal of the American Society of Nephrology*.

[41] Chung BH, Oh HJ, Piao SG (2012). Clinical significance of the ratio between FOXP3 positive regulatory T cell and interleukin-17 secreting cell in renal allograft biopsies with acute T-cell-mediated rejection. *Immunology*.

[42] Li Y, Shi Y, Huang Z (2011). CNI induced Th17/Treg imbalance and susceptibility to renal dysfunction in renal transplantation. *International Immunopharmacology*.

[43] Shao XS, Yang XQ, Zhao XD (2009). The prevalence of Th17 cells and FOXP3 regulate T cells (Treg) in children with primary nephrotic syndrome. *Pediatric Nephrology*.

